# Not everything is as it seems: neurosarcoidosis presenting as leptomeningitis

**DOI:** 10.1002/ccr3.1418

**Published:** 2018-02-13

**Authors:** Javier Andrés Galnares‐Olalde, Roberto Berebichez‐Fridman, Gilberto Gómez‐Garza, Moisés Mercado, Francisco Moreno‐Sánchez, Marco Antonio Alegría‐Loyola

**Affiliations:** ^1^ American British Cowdray Medical Center Mexico City Mexico; ^2^ School of Medicine Faculty of Health Sciences Anahuac University Mexico North Campus Huixquilucan Mexico

**Keywords:** Central nervous system, granulomatous disease, leptomeningitis, neurosarcoidosis, sarcoidosis, steroids

## Abstract

Involvement of the central nervous system in sarcoidosis is rare; neurosarcoidosis, although unusual, can present as leptomeningitis. The diagnosis is usually difficult because of the vague and broad symptomatology; therefore, a prompt diagnosis should be made, and adequate treatment should be administered to reduce morbidity and mortality.

## Introduction

Sarcoidosis, also known as Besnier‐Boeck or Besnier‐Boeck‐Schaumann disease, is a rare multisystemic inflammatory disease of unknown etiology and autoimmune behavior, which is characterized by the formation of noncaseating granulomas 1. Sarcoidosis can affect people of all ethnic and racial groups and may occur at all ages, although it typically develops earlier than the age of 50 years; its highest incidence is at 20–39 years. It has an estimated incidence of 5–40 cases per 100,000 people 1. The uppermost annual incidence has been reported in northern European countries, and it is more prone to be chronic and fatal in black Americans 1. It generally behaves as a silent disease that is diagnosed incidentally. The lung are the organ most frequently involved, although the heart, kidneys, skin, lymphatic system, liver, eyes, and central nervous system (SNC) may be also affected. The etiologies of sarcoidosis are unknown; nevertheless, there are hypotheses that environmental, genetic, and immunologic factors have a role in the development of the disease 1, 2. Sarcoidosis is constituted by the development and accumulation of granulomas, which are its fundamental abnormality 1.

Here, we present a rare case of neurosarcoidosis presenting as leptomeningitis, with radiographic, histopathological, and clinical evidence, in a 39‐year‐old woman that had a positive outcome after receiving adequate treatment. We also present a review of the literature in order to present the most recent and relevant information regarding neurosarcoidosis.

## Case Report

**Figure 1:**
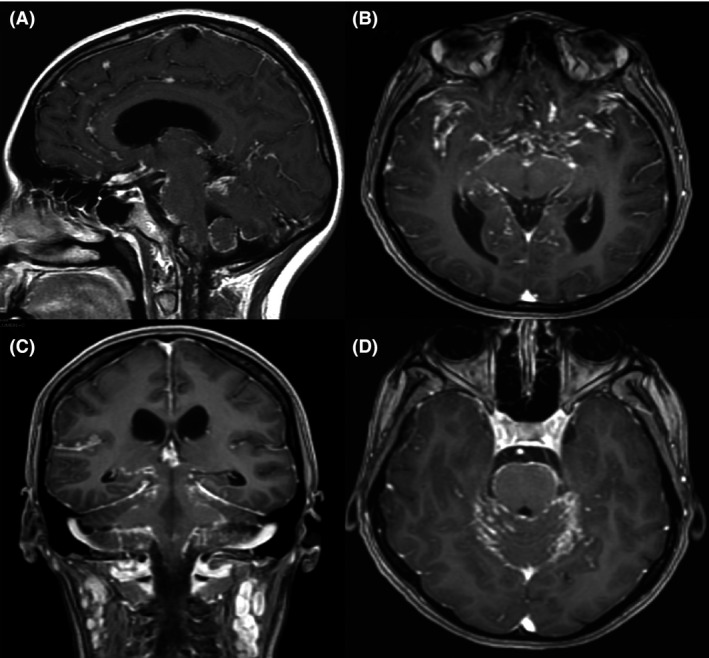
Brain Magnetic Resonance Imaging. T1‐weighted images after administration of gadolinium in the sagittal (A), coronal (C), and axial (B and D) planes. Generalized leptomeningeal reinforcement of basal predominance and multinodular appearance; hydrocephalus is also observed.

A 39‐year‐old woman without relevant medical history prior to admission presented to the emergency room with a 2‐week gradual onset of frontal headache, intermittent fever, nausea, vomiting, disorientation, and incoherent language. On examination, both pupils were miotic with an adequate response to light stimulus, without papilledema. Kernig, Brudzinski, and nuchal rigidity signs were positive. The rest of the neurological examination was normal, including strength, sensitivity, coordination, and balance. Complete blood count and liver function tests were normal, and while blood chemistry and hormone tests showed hyponatremia and low thyroid‐stimulating hormone (Table 1). A magnetic resonance imaging of the brain (MRI) was performed with and without the administration of contrast material, demonstrating generalized leptomeningeal reinforcement on T1‐weighted images of basal predominance and multinodular appearance, with evidence of hydrocephalus (Fig. 1). A lumbar puncture was also performed, and the cerebral spinal fluid (CSF) analyses reported polymorphonuclear‐predominating leukocytosis, hypoproteinorrachia, and hypoglycorrhachia. Gram stain was reported negative, and cultures were taken.

**Table 1:** Laboratory results of the patient in the first and second admissions and after 1‐year follow‐up

Variable	Reference range	First admission	Second admission	1‐year follow‐up
Hemoglobin (g/dL)	12–16.5	11.6	12	–
Hematocrit (%)	36–46–5	33.9	36.5	–
White‐cell count (per mm^3^)	4.8–10	5.3	4.5	–
Differential count (%)				
Neutrophils		76	57	–
Lymphocytes		18	25	
Monocytes		6	11	
Eosinophils		0	7	
Basophils		0	0	
Platelet count (per mm^3^)	150–450 × 10^3^	353 × 10^3^	281 × 10^3^	–
Mean corpuscular volume (fl)	80–99	86	89.2	–
Mean corpuscular hemoglobin (pg)	27–33	29	29.4	–
Sodium (mEq/L)	137–145	123	142	–
Potassium (mEq/L)	3.5–5.6	4.4	4	–
Chloride (mEq/L)	100–112	92	109	–
Carbon dioxide (mEq/L)	22–29	25.6	28.6	–
Calcium (mEq/L)	8.5–10.5	9.1	8.8	–
Phosphorus (mg/dL)	2.4–4.7	4.5	4.7	–
Magnesium (mg/dL)	1.9–2.5	1.8	1.9	–
Glucose (mg/dL)	60–100	105	89	–
Urea nitrogen (mg/dL)	6–20	12	5	–
Creatinine (mg/dL)	0.4–1.4	0.7	0.6	–
C‐reactive protein (mg/dL)	0–0.3	3.05	4.67	–
ESR (mm/h)	0–20	30	36	–
Procalcitonin (ng/mL)	<0.05	<0.05	<0.05	–
Serum cortisol (μg/dL)	3–16	169	2	2.7
Prolactin (ng/dL)	1.39–24.2	–	159	98.9
Luteinizing hormone (mUI/mL)	22–105	–	0.04	<0.10
Follicle‐stimulating hormone (mUI/mL)	9–16	–	1	1.93
Thyroid profile				
Thyroid‐stimulating hormone (μUI/mL)	0.45–5	0.37	0.9	0.10
Tyroxine (T4) (μg/dL)	4.5–12	5.3	3.7	2.9
Triyodotironin (T3) (ng/mL)	0.8–2	0.6	0.5	–
Liver function tests				
AST (U/L)	10–50	20	45	–
ALT (U/L)	8‐54	15	31	
Lactate dehydrogenase (U/L)	40–117	96	163	
Total bilirubin (mg/dL)	0.3–1–3	0.5	0.3	
Direct bilirubin (mg/dL)	0–0.4	0.2	0.2	
Indirect bilirubin (mg/dL)	0–0.8	0.3	0.1	
Total proteins (g/dL)	6.4–8.2	7.4	6.5	
Albumin (g/dL)	3.2–4.5	4	3.7	
Globulins (g/dL)	2.6–3.5	3.4	2.8	
Lumbar puncture				
Color	Rock water	Rock water	Rock water	–
Aspect	Transparent	Transparent	Transparent	
pH	7.4–7.7	8.1	8	
Erythrocytes (cells/mm^3^)	0–5	110	0	
Leucocytes (cells/mm^3^)	0–1	990	0	
Glucose (mg/dL)	45–80	10	27	
Proteins (mg/dL)	15–45	1	356	
DHL (U/L)	10–20	85	41	

A ventriculostomy was performed, and the patient was admitted to the intensive care unit for observation and initiation of empiric antibiotic therapy with meropenem, vancomycin, acyclovir, and prednisone for the clinical suspicion of SNC infection. Later on, CSF cultures reported no microbiological development. Hypernatremia behaved as diabetes insipidus and did not respond to fluid restriction. The patient improved neurologically, showing good response to the treatment, and was discharged 3 weeks after treatment initiation, with further consultation to endocrinology for the diminished thyroid levels and reducing‐dose steroid scheme until suspension.

**Figure 2:**
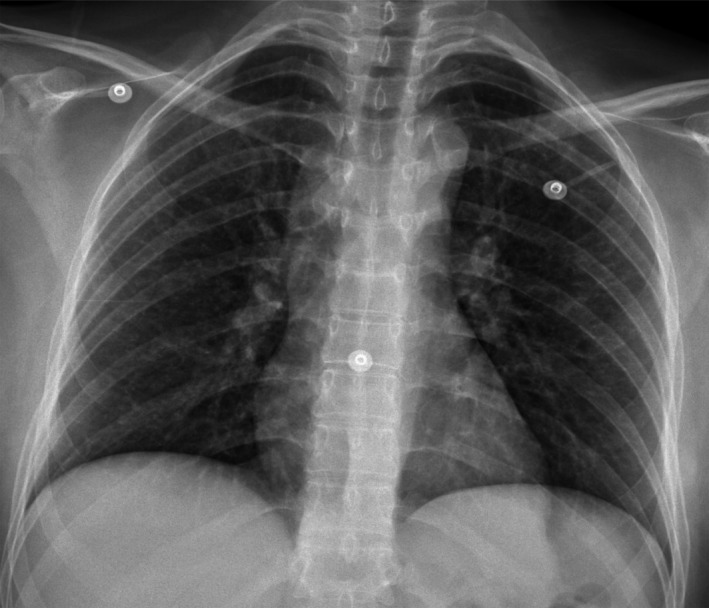
Chest radiography demonstrating the presence of a diffuse reticulonodular interstitial pulmonary pattern and a discrete mediastinal widening.

One month later, the patient initiated with the same neurological deficit as the previous admission, presenting also polyuria and polydipsia. A lumbar puncture was performed, showing only remarkable hyperproteinemia, without white blood cells (Table 1). A chest X‐ray demonstrated the presence of a diffuse interstitial pulmonary pattern and a discrete mediastinal widening (Fig. 2).

**Figure 3:**
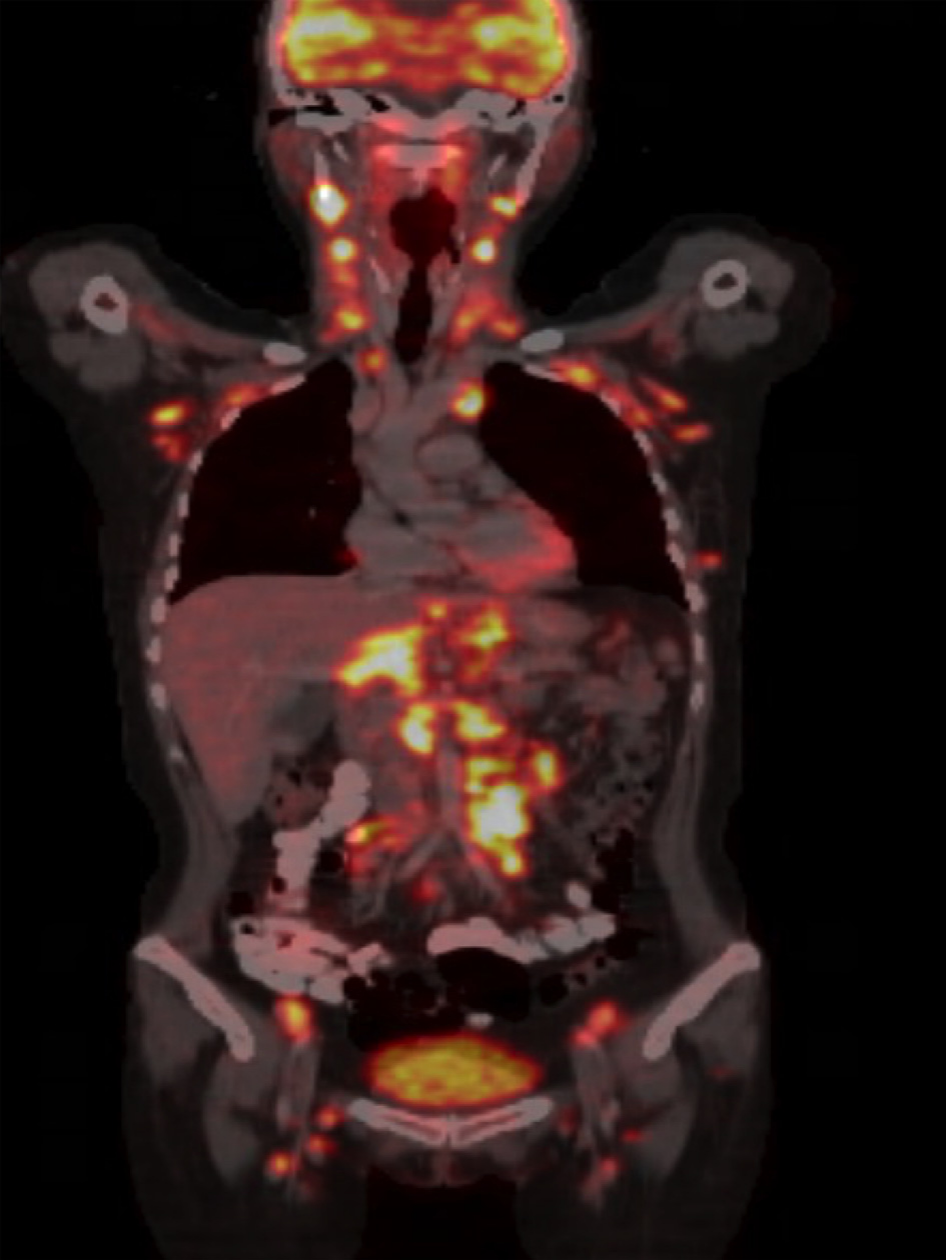
18 F‐FDG PET/CT coronal fusion demonstrating generalized enlargement of cervical, axillary, mediastinal, abdominal, and inguinal lymph nodes, with increased FDG uptake.

Because of the atypical recurrence of the neurological manifestations, including frontal headache, intermittent fever, nausea, vomiting, disorientation and incoherent language, and the CSF characteristics, a PET/CT was performed, demonstrating generalized enlargement of cervical, axillary, mediastinal, abdominal, and inguinal lymph nodes, with increased FDG uptake (Fig. 3). A supraclavicular lymph node biopsy was performed; the histopathological analyses reported the presence of noncaseating granulomas, compatible with sarcoidosis.

The patient was treated with intravenous Methylprednisolone with significant neurological improvement and resolution of the symptomatology, for which the patient was discharged. After 2 years of follow‐up, the patient presented with panhypopituitarism, which was managed by endocrinology with appropriate hormonal substitution and observation.

## Discussion

Involvement of the CNS in sarcoidosis is very unusual, occurring only in 5–25% of patients with sarcoidosis, although only 10% of all patients with sarcoidosis present with neurologic manifestations. Exclusive neurological forms of sarcoidosis occur in 5% of cases 2, 3. Neurosarcoidosis often presents when there is systemic involvement and it may comprise any part of the nervous system, which can have acute or chronic courses. The most common regions affected of the CNS are the hypothalamus, pituitary gland, and cranial nerves. The brainstem, spinal cord, parenchyma, and meninges are involved less commonly 2, 3.

The exact etiology of neurosarcoidosis is unknown and multifactorial, involving genetic predisposition and individual and environmental factors. Exposure to mildew, musty odors, pesticides, and agricultural employment have been associated with the development of sarcoidosis 1. Interestingly, tobacco has been negatively correlated with sarcoidosis. Protein antigens and mycobacterial DNA have been detected in sarcoid tissues and are believed to be the aim of T‐ and B‐cell responses, which suggests that microbial etiologies could participate in the development of neurosarcoidosis 2, 4. It is thought that neurosarcoidosis develops initially from a granulomatous inflammatory meningitis, which then extends from the Virchow‐Robin spaces in the subarachnoid space to the parenchyma 2.

Clinical manifestations of neurosarcoidosis can be found in 5–20% of cases of systemic sarcoidosis, and these symptoms can be mild or severe 5 (Table 2). About half of patients with neurosarcoidosis can present with neurologic manifestations sooner than systemic sarcoidosis is apparent. Some patients can present with hydrocephalous because of the development of granulomas on the fourth ventricle, which may require urgent treatment 1, 3.

**Table 2:** Clinical manifestations of systemic and isolated neurosarcoidosis [2, 3, 4, 5, 11.]

Clinical manifestations of neurosarcoidosis	
Neurological manifestations of systemic sarcoidosis (%)	Isolated neurosarcoidosis (%)
Peripheral neuropathy (69)	Headache (90)
Cranial neuropathy (66)	Paresthesias (50)
Depression (60)	Cranial neuropathy (40)
Headache (48)	Hemiparesia (30)
Hydrocephalus (38)	Weakness (30)
Radiculopathy (35)	Seizures (30)
Myopathy (rare)	Myelopathy (20)
Seizures (rare)	Radiculopathy (20)
	Cognitive alterations (20)
	Aseptic meningitis (10)
	Peripheral neuropathy (10)

Neurosarcoidosis can present as meningitis or panhypopituitarism, although it is extremely rare. Clinical course can present as a single episode, recurrent episodes, or as a chronic evolution of aseptic meningitis. Nonetheless, our patient had white blood cells with polymorphonuclear predominance, pattern suggestive of bacterial meningitis. Typically, pleocytosis can be seen in the cerebrospinal fluid examination, in association with myelopathy, cranial neuropathy, mass lesion, or other focal neurologic findings 2, 5. One of the most important symptoms of patients with neurosarcoidosis is headache, which may resemble the pain from meningitis. Patients can also present with generalized, complex partial, and myoclonic seizures in about 5–22% of the cases. Involvement of the pituitary gland is a common feature of neurosarcoidosis, especially the posterior pituitary. Patients can present with polydipsia and polyuria resultant from diabetes insipidus 6. Other manifestations are weight gain, amenorrhea, hypothyroidism, personality changes, temperature dysregulation, galactorrhea, and sleep disorders 2, 4. Involvement of the basal ganglia can cause hemichoreoathetosis 7.

Patients with neurosarcoidosis can also have neuropsychiatric manifestations, such as dementia, psychoses, depression, poor concentration, encephalopathy, and hallucinations. Cranial and peripheral neuropathies may be present in cases of neurosarcoidosis 8. Facial palsy is the most frequent cranial neuropathy, although involvement of the trigeminal nerve has been reported 8. Neurosarcoidosis can be associated with uveitis, parotid gland swelling, and chronic fever, known as Heerfordt Syndrome 5. In up to 38% of patients, the optic nerve may be compromised and may present as a painless and progressive monocular visual loss, which may be accompanied by optic atrophy and papilledema. Vestibular dysfunction and hearing loss due to VIII nerve neuropathy can also be present. Lower cranial nerves may also be involved, causing dysarthria or dysphagia 2, 4, 9.

Sarcoidosis may cause involvement of peripheral nerves in up to 20% of patients. The most common presentation is symmetrical distal neuropathy, followed by mononeuropathy, acute or chronic demyelinating polyneuropathy, and mononeuritis multiplex 4. Due to the multiple clinical manifestations that patients with neurosarcoidosis may have, the diagnostic workup should be complete and multidisciplinary. Neurosarcoidosis should be suspected in patients with diagnosed sarcoidosis that show neurologic symptomatology, and the diagnosis can be performed according to the modified Zajicek criteria (Table 3), although there are several other diagnostic criteria 4, 5, 10, 11. Other diseases capable of producing noncaseating granulomas should be excluded. It is imperative to establish if sarcoidosis arises as a systemic disease or if it is constrained to the CNS. A chest radiography and computed tomography should be obtained. Also, pulmonary function tests should be performed which may reveal intrathoracic pathology 2. Granulomas found in sarcoidosis generate angiotensin‐converting enzyme (ACE), and levels of this enzyme are elevated in 60% of patients with sarcoidosis. This test is insensitive, nonspecific, and a poor therapeutic guide; its value in diagnosing and managing sarcoidosis remains controversial 12. Bronchoalveolar lavage (BAL) can be employed as a diagnostic aid to support the diagnosis of sarcoidosis, which demonstrates a cell pattern characteristic of lymphocytic alveolitis. It is known that the ACE levels in BAL have a higher prognostic value than serum ACE 12. Histopathology evaluation from a biopsy that shows noncaseating granulomas is the best method for the diagnosis of sarcoidosis 1, 2, 4. Patients with suspected neurosarcoidosis should undergo MRI of the brain and spine 5, 13. Gadolinium is useful to evaluate leptomeningeal involvement, cranial nerve lesions, and parenchymal abnormalities 5, 13. The lesions can appear hyperintense in T2‐weighted MRI images, which are commonly localized in the superficial and deep white matter. These imaging features may be similar to those seen in multiple sclerosis 5, 13. It is imperative to mention that ancillary investigations for neurosarcoidosis have a low sensitivity, as noted by Fritz et al.; therefore, the diagnosis should be complemented with clinical findings and histopathology 14.

**Table 3:** Modified Zajicek criteria for the diagnosis of neurosarcoidosis

Possible	There are clinical and neuroradiologic data suggestive of neurosarcoidosis. Malignancy and infection have not been rigorously excluded or there is no pathologic confirmation of systemic sarcoidosis
Probable	There are clinical and neuroradiologic data suggestive of neurosarcoidosis. Malignancy and infection have been excluded, and there is pathologic evidence of sarcoidosis.
Definitive	The criteria of probable diagnosis + response to therapy for neurosarcoidosis over a one‐ to two‐year observation period.

Analysis of the CSF can be helpful, although it is not specific 4. Those patients with meningeal disease may have hypoglycorrhachia as low as 30 mg/dL, high opening pressure, and mononuclear pleocytosis ranging from 10 to 100 cells/mm^3^. ACE in the CSF may be particularly helpful in the monitoring of treatment response and disease activity for patients with neurosarcoidosis 4. Wengert et al. made an interesting study, to detect whether the CSF parameters may be related to the MRI findings and the clinical disease activity. They detected that patients with diffuse leptomeningeal gadolinium enhancement on MRI had significantly higher cell counts, total protein, CSF/serum albumin quotients, and lactate; nevertheless, these patients also had significantly lower glucose levels, in comparison with patients without leptomeningeal enhancement. Patients with clinically active disease had significantly higher CSF cells counts, total protein, CSF/serum albumin quotients, and lactate, but significantly lower glucose levels than patients with stable disease. These findings support that CSF abnormalities in neurosarcoidosis are most pronounced in patients with diffuse leptomeningeal enhancement on MRI and that CSF analyses may assist in the distinction of different radiographic and pathologic manifestations of neurosarcoidosis. Moreover, CSF analyses may allow monitoring the disease activity in patients with neurosarcoidosis 15.

As neurosarcoidosis may present with a great amount of unspecific symptoms, the differential diagnosis is very broad and it depends on the clinical presentation 1, 2, 4. It is crucial to rule out infectious diseases, malignancy, and other autoimmune inflammatory diseases. The most common differential diagnoses are tumors such as lymphoma, meningioma carcinomatous meningitis, and gliomas; infections including neurosyphilis, HIV, tuberculosis, herpes family viruses, toxoplasmosis, and others such as multiple sclerosis, Guillain–Barré syndrome, systemic lupus erythematosus, amyloidosis, and porphyria 1, 2, 4.

Manifestations such as aseptic meningitis and isolated cranial nerve abnormalities usually have good response to short steroid course. On the other hand, patients who have mass lesions, hydrocephalus, leptomeningeal involvement, spinal cord disease, seizures, parenchymal disease, and multiple cranial nerve involvement usually require an elevated dose and extended course of steroids. These patients should be selected for early aggressive immunosuppressive therapy 2, 4. According to some reports, amid patients who receive steroids only, 35% improved on this treatment, and about 69% improved with immunosuppressive agents 2, 4.

Steroids can be started at 1 mg/kg or as a pulse of methylprednisolone, followed by gradual reduction. For patients with facial palsy, neuropathy, or myopathy, steroids can be started at 0.5 mg/kg for two to four weeks, followed by gradual decrease. Patients should have a close follow‐up to detect adverse effects from steroids. To diminish the risks of developing adverse effects, patients should be maintained on doses of steroids of <10 mg/day 1, 4, 9. Other commonly prescribed and effective drugs for treating neurosarcoidosis that have been reported are methotrexate, azathioprine, and mycophenolate 2, 4, 6. For refractory cases, cyclophosphamide, Infliximab, and Etanercept may be used with caution 4, 16, 17.

Radiotherapy for neurosarcoidosis can be used when medical therapy causes insupportable side effects or fails. Radiotherapy is effective in the prevention of progression of local symptoms of neurosarcoidosis and for sarcoid meningitis 2. Although new treatments have emerged, approximately one‐third of patients do not respond to treatment, even after the administration of second‐ and third‐line medications 14.

## Conclusions

Involvement of the CNS in sarcoidosis is rare, and neurosarcoidosis often presents when there is systemic involvement. The diagnosis should be made based on the clinical and radiographic findings and must be confirmed with a biopsy. The treatment will differ depending on the clinical manifestations, but the most common treatment is the use of steroids and other immunosuppressive drugs.

More research needs to be performed to better comprehend the etiology and pathophysiology of sarcoidosis. It is imperative to perform a prompt diagnosis so a timely treatment can be provided, thus reducing the morbidity and mortality associated with this disease.

## Authorship

MM, FMS, and MAAL: were involved in the patient's care at the American British Cowdray Medical Center. JAGO and RBF: wrote and reviewed the manuscript. GGG reviewed all MRI images and reports. All authors were involved in the editing and final approval of the manuscript.

## Ethical Statement

Witten consent was obtained from the patient for the publication of this case report and any accompanying images.

## Conflict of Interest

The authors declare no competing interests.
